# Temporal trends in the use of targeted temperature management after cardiac arrest and association with outcome: insights from the Paris Sudden Death Expertise Centre

**DOI:** 10.1186/s13054-019-2677-1

**Published:** 2019-12-03

**Authors:** Jean-Baptiste Lascarrou, Florence Dumas, Wulfran Bougouin, Richard Chocron, Frankie Beganton, Stephane Legriel, Nadia Aissaoui, Nicolas Deye, Lionel Lamhaut, Daniel Jost, Antoine Vieillard-Baron, Eloi Marijon, Xavier Jouven, Alain Cariou, on behalf of the SDEC, F. Adnet, J. M. Agostinucci, N. Aissaoui-Balanant, V. Algalarrondo, F. Alla, C. Alonso, W. Amara, D. Annane, C. Antoine, P. Aubry, E. Azoulay, F. Beganton, D. Benhamou, C. Billon, W. Bougouin, J. Boutet, C. Bruel, P. Bruneval, A. Cariou, P. Carli, E. Casalino, C. Cerf, A. Chaib, B. Cholley, Y. Cohen, A. Combes, M. Crahes, D. Da Silva, V. Das, A. Demoule, I. Denjoy, N. Deye, G. Dhonneur, J. L. Diehl, S. Dinanian, L. Domanski, D. Dreyfuss, D. Duboc, J. L. Dubois-Rande, F. Dumas, J. P. Empana, F. Extramiana, M. Fartoukh, F. Fieux, M. Gabbas, E. Gandjbakhch, G. Geri, B. Guidet, F. Halimi, P. Henry, F. Hidden Lucet, P. Jabre, L. Jacob, L. Joseph, D. Jost, X. Jouven, N. Karam, H. Kassim, J. Lacotte, K. Lahlou-Laforet, L. Lamhaut, A. Lanceleur, O. Langeron, T. Lavergne, E. Lecarpentier, A. Leenhardt, N. Lellouche, V. Lemiale, F. Lemoine, F. Linval, T. Loeb, B. Ludes, C. E. Luyt, A. Maltret, N. Mansencal, N. Mansouri, E. Marijon, J. Marty, E. Maury, V. Maxime, B. Megarbane, A. Mekontso-Dessap, H. Mentec, J. P. Mira, X. Monnet, K. Narayanan, N. Ngoyi, M. C. Perier, O. Piot, R. Pirracchio, P. Plaisance, I. Plu, M. Raux, F. Revaux, J. D. Ricard, C. Richard, B. Riou, F. Roussin, F. Santoli, F. Schortgen, A. Sharifzadehgan, G. Sideris, C. Spaulding, J. L. Teboul, J. F. Timsit, J. P. Tourtier, P. Tuppin, C. Ursat, O. Varenne, A. Vieillard-Baron, S. Voicu, K. Wahbi, V. Waldmann

**Affiliations:** 1 Service de Medecine Intensive Reanimation, Centre Hospitalier Universitaire, 30 Boulevard Jean Monnet, 44093 Nantes Cedex 9, France; 2 Université de Paris, INSERM, Paris Cardiovascular Research Centre, Paris, France; 3 Paris Sudden Death Expertise Center, Paris, France; 4 AfterROSC Network Group, Paris, France; 5 Emergency Department, Cochin University Hospital, APHP, Paris, France; 6 Medical Surgical Intensive Care Unit, Mignot Hospital, Le Chesnay, France; 7 Medical Intensive Care Unit, European University Hospital, Paris, France; 8 Medical Intensive Care Unit, Lariboisière University Hospital, Paris, France; 9 SAMU de Paris, DAR Necker University Hospital-Assistance, Paris, France; 10 Brigade des Sapeurs-Pompiers de Paris, Paris, France; 11 Medical Intensive Care Unit, Ambroise Pare University Hospital, APHP, Boulogne-Billancourt, France; 12 Medical Intensive Care Unit, Cochin University Hospital, APHP, Paris, France

**Keywords:** Cardiac arrest, Induced hypothermia, Targeted temperature management, Neurological outcome

## Abstract

**Purpose:**

Recent doubts regarding the efficacy may have resulted in a loss of interest for targeted temperature management (TTM) in comatose cardiac arrest (CA) patients, with uncertain consequences on outcome. We aimed to identify a change in TTM use and to assess the relationship between this change and neurological outcome.

**Methods:**

We used Utstein data prospectively collected in the Sudden Death Expertise Center (SDEC) registry (capturing CA data from all secondary and tertiary hospitals located in the Great Paris area, France) between May 2011 and December 2017. All cases of non-traumatic OHCA patients with stable return of spontaneous circulation (ROSC) were included. After adjustment for potential confounders, we assessed the relationship between changes over time in the use of TTM and neurological recovery at discharge using the Cerebral Performance Categories (CPC) scale.

**Results:**

Between May 2011 and December 2017, 3925 patients were retained in the analysis, of whom 1847 (47%) received TTM. The rate of good neurological outcome at discharge (CPC 1 or 2) was higher in TTM patients as compared with no TTM (33% vs 15%, *P* < 0.001). Gender, age, and location of CA did not change over the years. Bystander CPR increased from 55% in 2011 to 73% in 2017 (*P* < 0.001) and patients with a no-flow time longer than 3 min decreased from 53 to 38% (*P* < 0.001). The use of TTM decreased from 55% in 2011 to 37% in 2017 (*P* < 0.001). Meanwhile, the rate of patients with good neurological recovery remained stable (19 to 23%, *P* = 0.76). After adjustment, year of CA occurrence was not associated with outcome.

**Conclusions:**

We report a progressive decrease in the use of TTM in post-cardiac arrest patients over the recent years. During this period, neurological outcome remained stable, despite an increase in bystander-initiated resuscitation and a decrease in “no flow” duration.

## Introduction

Over the last decades, implementation of the “chain of survival” led to a progressive improvement in outcome after cardiac arrest (CA) in many places across the world [1]. In addition to pre-hospital management, post-cardiac arrest care is now considered as a major determinant of outcome. Among the interventions that can be provided during the post-resuscitation period, recent data raised some concerns regarding the benefit of targeted temperature management (TTM) for comatose CA survivors. Even if TTM is strongly recommended in out-of-hospital CA provoked by a ventricular arrhythmia, its effectiveness has been seriously challenged in several other situations, such as in non-shockable [2] and in-hospital CA patients [3]. Additionally, the TTM trial that showed no difference between 33 and 36° may have been falsely considered by some clinicians as a negative study regarding the effectiveness of TTM [4]. On the whole, this may have resulted in a change in TTM modalities in post-cardiac patients, as reported by several investigators [5]. In a recent survey, Deye et al. observed that 37% of responders changed their attitude regarding TTM over the recent years, many of them moving from 33 °C toward a higher temperature level [6].

Possible consequences of these changes on patients’ outcome are unclear. After moving from a TTM target of 33 to 36 °C, Bray et al. reported low compliance with target temperature, higher rates of fever, and a trend toward clinical worsening in patient outcomes [7]. In a recent retrospective study performed in Australia and New Zealand, similar changes in clinicians’ attitude regarding target temperature after CA, which occurred in up to one third of ICU, were associated with an increased proportion of patients with fever [5]. In the USA, where this treatment was historically less employed as compared with European countries, TTM use was shown to further decrease in many centers, as revealed by recent administrative data [8]. In parallel, Khera et al. found that TTM use dropped in all subgroups of CA survivors (both shockable and non-shockable) [9]. On the whole, these uncertainties prompted to perform new large randomized trials testing this treatment in CA patients. The recently released HYPERION study showed an improvement in long-term neurological prognosis with TTM at 33° as compared to normothermia for CA patients with non-shockable rhythm [10], and the TTM2 trial comparing TTM at 33° versus fever control is actually ongoing.

We hypothesized that a change in French clinicians’ attitude regarding TTM may have occurred in the period that preceded these new trials and that this change may have affected the outcome in post-CA patients. In order to explore this hypothesis, we decided to perform an in-depth analysis of a regional registry covering the Great Paris area (France).

## Materials and methods

### Study design

We used data extracted from the cardiac arrest registry managed by the Sudden Death Expertise Center (SDEC) of the Great Paris area (France), which was previously described [11]. Our aim was to search for a change in the use of TTM over the recent years and to assess the relationship between this change and the patient’s outcome after adjustment for potential confounders.

### Study setting

In Paris and its surrounding suburbs (Haut-de-Seine, Seine-Saint-Denis, Val-de-Marne), the management of OHCA involves mobile emergency units and fire departments, covering 762 km^2^ and a population of 6.6 million inhabitants. The Emergency Medical Service is a two-tiered physician-manned system, with a basic life support (BLS) tier served by firefighters of the Brigade de Sapeurs-Pompiers de Paris, who can apply automated external defibrillators, and an advanced cardiac life support (ACLS) tier, provided in the field, with endotracheal intubation, intravenous access line, and drugs if necessary. Resuscitation is delivered by an emergency team that includes at least one trained physician in emergency medicine and one nurse, applying international guidelines. Patients with stable return of spontaneous circulation (ROSC) are then transferred to a secondary or tertiary center with an intensive care unit (ICU) and coronary intervention facilities with a target of door to balloon of 120 min maximum for acute coronary syndrome with ST elevation on ECG.

From 2011, all OHCA cases occurring in Paris and its suburbs are recorded in a prospective population-based registry system managed by the Paris Sudden Death Expertise Center (Paris-SDEC) [12, 13].

### Study population

According to recent guidelines [14], all cases of OHCA (defined as unexpected death without obvious extra-cardiac cause, such as drowning, trauma, hanging, intoxication) are prospectively recorded in the Paris-SDEC registry according to Utstein style. In the present analysis, we retained all post-cardiac arrest patients transported to the hospital and admitted in ICU with a sustainable ROSC from May 15, 2011, to December 31, 2017. Exclusion criteria were age below 18 years, prior terminal condition (e.g., metastatic malignancy), obvious non-cardiac cause according to Utstein templates [14], patients who died before hospital admission, and refractory OHCA transported to the hospital for potential extra-corporal life support (ECLS).

### Data collection

The following variables are collected in the registry: age, gender, location, witnessed status, bystander cardiopulmonary resuscitation, initial cardiac rhythm, cumulative epinephrine dose employed during resuscitation, delays from collapse to start of chest compressions (no flow) and from start of chest compression to ROSC (low flow), characteristics of the post-resuscitation ECG, arterial pH at hospital admission, temperature management, and coronary interventions after hospital admission.

The neurological outcome was scored using the level reached on the Cerebral Performance Categories (CPC) scale [15] at ICU discharge, assessed by the physician in charge. Patients with a good cerebral performance (CPC1) or a moderate cerebral disability (CPC2) were considered to have a favorable neurological recovery.

### Patients’ management

TTM was considered as the provision of any measure aiming to reduce the patient’s body temperature by either non-invasive or by invasive means. According to current guidelines, the targeted temperature was comprised between 32 and 36 °C [16]. TTM could be started in the emergency department or in the ICU according to each center policy.

Early invasive coronary strategy was defined as a coronary angiography (followed by percutaneous coronary intervention if indicated) performed in the very first hours following hospital admission. Post-resuscitation shock was defined as the occurrence or persistence of arterial hypotension (mean arterial pressure < 60 mmHg or systolic blood pressure < 90 mmHg) sustained for more than 6 h after ROSC despite adequate fluid resuscitation and requiring a continuous infusion of vasopressor.

Definitions and modalities for data collection were unchanged during the period of the study. Data were entered prospectively into a study database and checked for completeness and accuracy. Two investigators (FD, AC) reviewed each record for data completion and validity.

### Statistical analysis

We used descriptive statistics to summarize categorical variables as proportions, and continuous variables as mean with standard deviation for normal distribution or as median with interquartile range for non-normal distribution. Comparisons between proportions used Pearson’s chi-squared (or Fisher’s exact test if appropriate) and *t* test for continuous variables (or Mann-Wilcoxon rank-sum test).

According to the different analyses, we used three different options regarding the “year of cardiac arrest,” which was analyzed as a continuous or a categorical variable, or divided into four a priori defined periods (P) based on the publication of the TTM trial [3]: P1 was the baseline period (year 2011); P2 was the period before the publication of the TTM trial (years 2012 and 2013); P3 was the period that immediately followed the publication of the TTM trial (year 2014), and P4 being the most recent period (years 2015, 2016, and 2017).

We first assessed the relationship between baseline characteristics and the use of TTM. Test for trend and logistic regression were used to evaluate the pattern of neurological recovery over time and the potential explicative factors.

We then performed a univariate analysis testing the different parameters associated with good neurological recovery at ICU discharge. Association between TTM and outcome was analyzed in a multivariate model adjusted on Utstein variables including age, gender, location of arrest, provision of bystander CPR, resuscitation intervals, initial rhythm, epinephrine use, arterial pH at admission, occurrence of a post-cardiac arrest shock, early coronary angiogram performance, TTM, and year of arrest as continuous. In order to take into account missing data, we performed multiple imputations using chained equation [17] on the dataset restricted to patients with available survival status at hospital discharge (primary outcome), based on M = 10 imputed completed datasets.

In a *sensitivity* analysis aiming to assess the influence of prognostic factors over time, we included in the primary multivariate model an interaction (cross-product) term between year and covariates of interest (initial rhythm, bystander CPR performance, TTM).

Focusing on the role of TTM, we also evaluated the potential for generalized temporal trends to account for the study period association by using segmented regression analysis of interrupted time series data using time categorized in four a priori sub-periods (P1, P2, P3, and P4). This method allows to assess how much an intervention changed an outcome of interest, immediately and over time [18].

All tests were two-sided with a *P* value considered significant if < 0.05. Analyses were performed using STATA/SE 14.2 (Lakeway Drive, TX, USA).

## Results

During the study period, 25,059 patients were included in the Paris-SDEC registry. Cardiopulmonary resuscitation was not attempted in 7923 patients, an obvious non-cardiac cause of arrest was evidenced in 3782 patients, resuscitation failed in 8766 patients (who were not transported according to French EMS policy), and 663 patients with refractory CA were transported without ROSC to the hospital for potential extra-corporeal life support. This left 3925/25059 (16%) patients suitable for the present analysis, of whom 1847/3925 (47%) received TTM (see Additional file 1). Numbers of missing data are displayed on Additional file 2.

Patients’ characteristics according to temperature management are displayed in Table 1. As compared with controls, those who received TTM were more frequently men and younger; cardiac arrest occurred more frequently in a public place, and the proportion of patients with a long no-flow duration (higher than 3 min) was greater (*P* < 0.001). An early invasive coronary strategy was used more frequently in TTM patients (*P* < 0.001). As compared with controls, those who received TTM had more frequently a good neurological recovery at ICU discharge (15% vs 33%, *P* < 0.001).

**Table 1:** Characteristics of the study population according to targeted temperature management

	No TTM (*n* = 1793)	TTM (*n* = 1847)	*P*
Male, *n* %	1169 (65%)	1318 (71%)	< 0.001
Age, ±SD	64 ± 16	61 ± 15	< 0.001
Home location, *n* %	1186 (66%)	1017 (55%)	< 0.001
Witness, *n* %	1569 (87%)	1697 (92%)	< 0.001
Bystander CPR, *n* %	1142 (63%)	1195 (65%)	< 0.001
Shockable rhythm, *n* %	592 (33%)	1065 (58%)	< 0.001
No-flow duration > 3 min*, *n* %	666 (37%)	881 (48%)	< 0.001
Low-flow duration > 20 min*, *n* %	588 (33%)	760 (42%)	< 0.001
Epinephrine use, *n* %	1259 (70%)	1224 (66%)	< 0.001
First arterial pH, ±SD	7.13 ± 0.22	7.22 ± 0.15	< 0.001
Post-resuscitation shock, *n* %	863 (48%)	1253 (68%)	< 0.001
Early invasive coronary strategy, *n* %	767 (43%)	1494 (80%)	< 0.001
Survival at ICU discharge, *n* %	370 (20%)	689 (37%)	< 0.001
Good neurological prognosis at ICU discharge, *n* %	273 (15%)	618 (33%)	< 0.001

Values are expressed with proportion (%), mean with standard deviation (SD)

*CPR* cardiopulmonary resuscitation

*Characteristics were dichotomized according to the median value

**Fig. 1:**
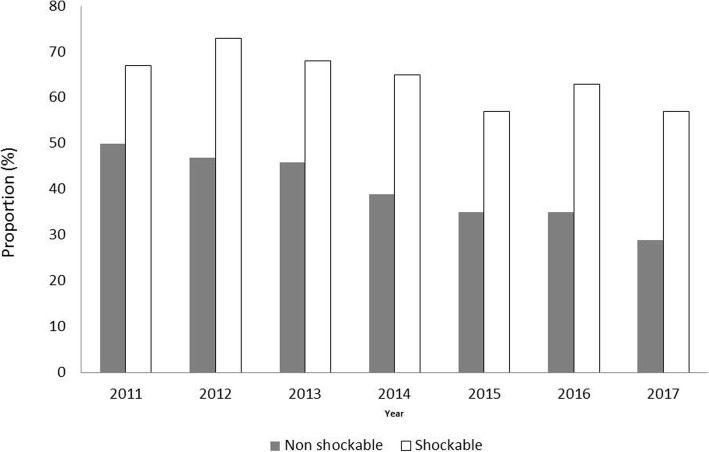
Changes in TTM use over time according to initial cardiac rhythm

Changes over the years in patients’ characteristics and management appear in Table 2. Gender, age, and location of CA did not significantly change over time. Bystander CPR increased from 55% in 2011 to 73% of patients in 2017 (*P* < 0.001), no-flow time upper than 3 min decreased from 53 to 38% (*P* < 0.001) (Table 2). During the study period, the overall proportion of patients receiving TTM decreased from 55% in 2011 to 37% in 2017 (*P* < 0.001). This decrease in TTM use over years was observed in both shockable and non-shockable patients (see Fig. 1). Using segmented regression separating the study period into four separate sub-periods (P1 = 2011, P2 = 2012–2013, P3 = 2014, P4 = 2015–17), the decrease in TTM use was significant for P3 vs. P2 (OR = 0.78 [0.64–0.96] *P* = 0.02) and P4 vs. P3 (OR = 0.77 [0.63–0.94] *P* = 0.009). In parallel, the survival rate at ICU discharge increased significantly from 20% in 2011 to 26% in 2017 (*P* = 0.03), but the proportion of patients with good neurological outcome remained stable (19 to 23%; *P* = 0.76) (Fig. 3).

**Table 2:** Baseline characteristics evolution over the years

	All	2011	2012	2013	2014	2015	2016	2017	*P* for trend
*N*	3925	339	562	589	594	559	620	662	
Male, *n* (%)	2682 (70%)	235 (70%)	386 (68%)	395 (67%)	401 (67%)	394 (70%)	442 (71%)	429 (65%)	0.63
Age, mean ± SD	62 ± 15	62 ± 14	62 ± 15	62 ± 15	62 ± 16	61 ± 15	63 ± 15	62 ± 16	0.17
Home location, *n* (%)	2365 (58%)	206 (60%)	341 (60%)	338 (57%)	342 (57%)	338 (60%)	392 (63%)	408 (61%)	0.17
Witnessed, *n* (%)	3504 (90%)	320 (94%)	502 (89%)	516 (88%)	534 (90%)	489 (87%)	543 (88%)	600 (90%)	0.44
Bystander CPR, *n* (%)	2525 (66%)	186 (55%)	310 (55%)	359 (60%)	384 (65%)	365 (65%)	437 (70%)	484 (73%)	< 0.001
Initial shockable rhythm, *n* (%)	2215 (50%)	130 (38%)	256 (45%)	273 (46%)	298 (50%)	251 (45%)	289 (46%)	287 (43%)	0.26
No flow > 3 min*, *n* (%)	1784 (40%)	179 (53%)	231 (41%)	236 (40%)	248 (42%)	232 (41%)	234 (38%)	253 (38%)	< 0.001
Low flow > 20 min*, *n* (%)	1613 (39%)	121 (36%)	203 (36%)	207 (35%)	185 (31%)	198 (35%)	219 (35%)	264 (40%)	0.039
Use of epinephrine, *n* (%)	2646 (70%)	245 (72%)	373 (66%)	401 (68%)	401 (67%)	393 (70%)	396 (64%)	437 (66%)	0.10
First arterial pH, mean ± SD	7.2 ± 0.2	7.2 ± 0.2	7.2 ± 0.2	7.2 ± 0.2	7.2 ± 0.2	7.2 ± 0.2	7.2 ± 0.2	7.2 ± 0.2	0.49
Post-resuscitation shock, *n* (%)	2149 (56%)	253 (74%)	388 (69%)	338 (57%)	270 (45%)	305 (54%)	318 (51%)	277 (42%)	< 0.001
Early invasive coronary strategy, *n* (%)	2349 (60%)	202 (60%)	336 (60%)	354 (60%)	367 (62%)	335 (60%)	388 (63%)	367 (55%)	0.13
Targeted temperature management, *n* (%)	1847 (47%)	189 (55%)	321 (57%)	311 (53%)	275 (46%)	230 (41%)	278 (45%)	243 (37%)	< 0.001
Survival at ICU discharge, *n* (%)	1106 (26%)	70 (20%)	153 (27%)	181 (30%)	188 (32%)	161 (29%)	176 (28%)	177 (26%)	0.03
Good neurological prognosis at ICU discharge, *n* (%)	921 (23%)	63 (19%)	141 (26%)	160 (28%)	151 (27%)	135 (26%)	147 (26%)	124 (23%)	0.76

Values are expressed with proportion (%), mean with standard deviation (SD)

*CPR* cardiopulmonary resuscitation

*Characteristics were dichotomized according to the median value

In univariate analysis, TTM was significantly associated with a better neurological outcome with OR = 2.71 [2.30–3.19] and was associated with a better outcome after adjustment on baseline characteristics and year of arrest (adjOR = 1.57 [1.22–2.02]). Considering 2011 as the year of reference, a later onset of CA was not associated with a better outcome in univariate analysis (*P* = 0.75) (Table 3). In addition, no interaction term between year of occurrence and parameters of interest (initial rhythm, bystander CPR performance, TTM) was significant (*P* for interaction > 0.10).

**Table 3:** Factors associated with good neurological prognosis at ICU discharge in uni- and multivariate analysis in multiple imputation cohort (*N* = 3623 patients)

Characteristics	Univariate analysis		Multivariate analysis	
	Coefficient [95%CI]	*P* value	Coefficient [95%CI]	*P* value
Male	1.65 [1.39–1.97]	< 0.001	–	–
Age	0.97 [0.96–0.98]	< 0.001	0.96 [0.95–0.97]	< 0.001
Home location	0.32 [0.27–0.38]	< 0.001	0.59 [0.47–0.74]	< 0.001
Bystander CPR	2.62 [2.16–3.19]	< 0.001	1.65 [1.19–2.28]	0.003
Shockable rhythm	9.70 [8.04–11.70]	< 0.001	2.96 [2.29–3.93]	< 0.001
No flow > 3 min*	0.38 [0.32–0.45]	< 0.001	0.48 [0.36–0.64]	< 0.001
Low flow > 20 min*	0.21 [0.17–0.26]	< 0.001	0.50 [0.38–0.66]	< 0.001
Epinephrine use*	0.06 [0.05–0.08]	< 0.001	0.15 [0.11–0.19]	< 0.001
First arterial pH (by 0.1 unit)	1.86 [1.73–2.00]	< 0.001	1.31 [1.20–1.45]	< 0.001
Post-resuscitation shock	0.44 [0.37–0.42]	< 0.001	0.73 [0.57–0.94]	0.012
Early invasive coronary strategy	8.16 [6.50–10.25]	< 0.001	3.24 [2.37–4.43]	< 0.001
Targeted temperature management	2.71 [2.30–3.19]	< 0.001	1.57 [1.22–2.02]	< 0.001
Year of occurrence (2011 as reference)	1.01 [0.96–1.05]	0.75	–	–

*CPR* cardiopulmonary resuscitation

*Characteristics were dichotomized according to the median value

**Fig. 2:**
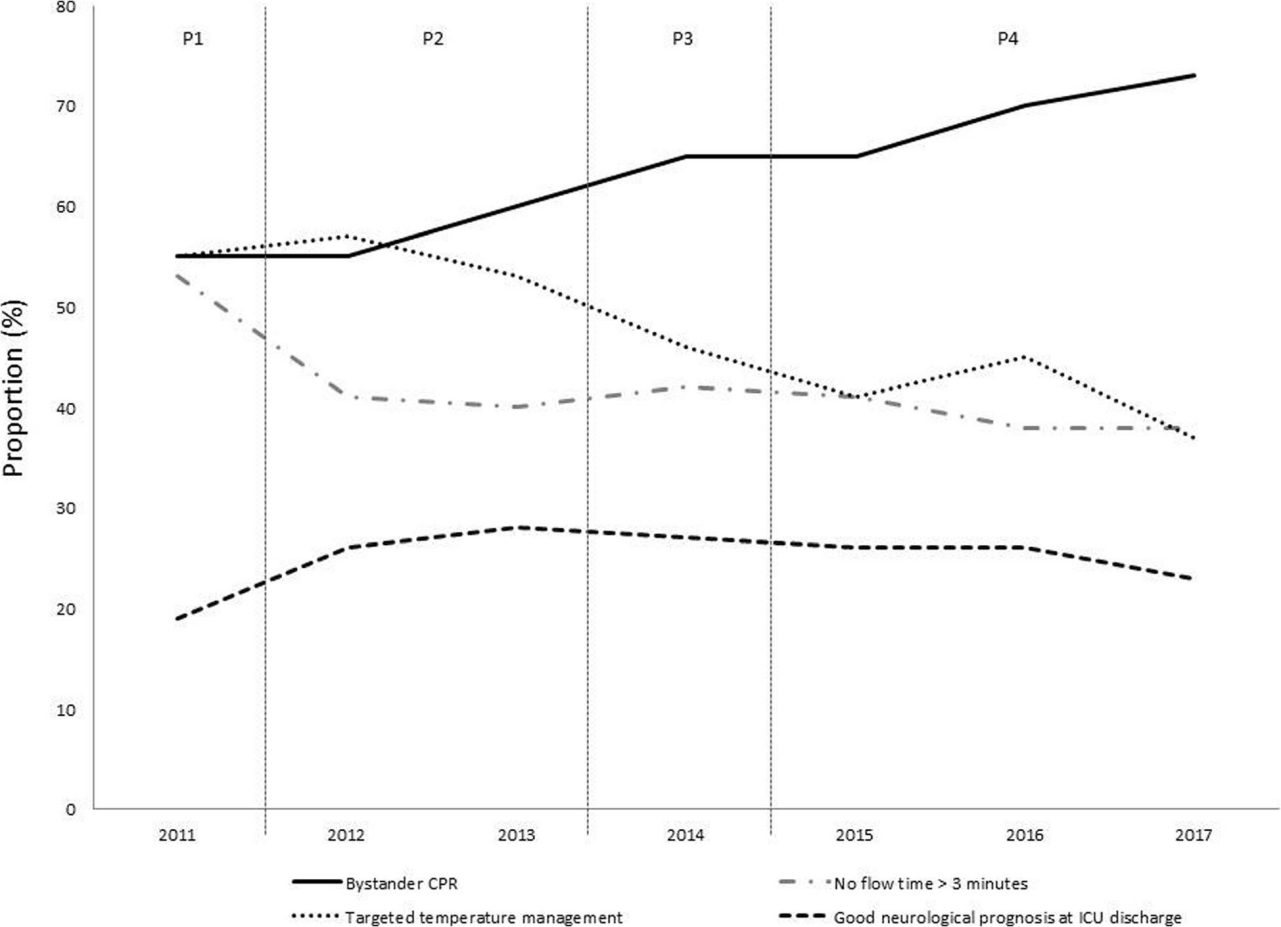
Changes in characteristics and outcome according to periods of segmented regression analysis

**Fig. 3:**
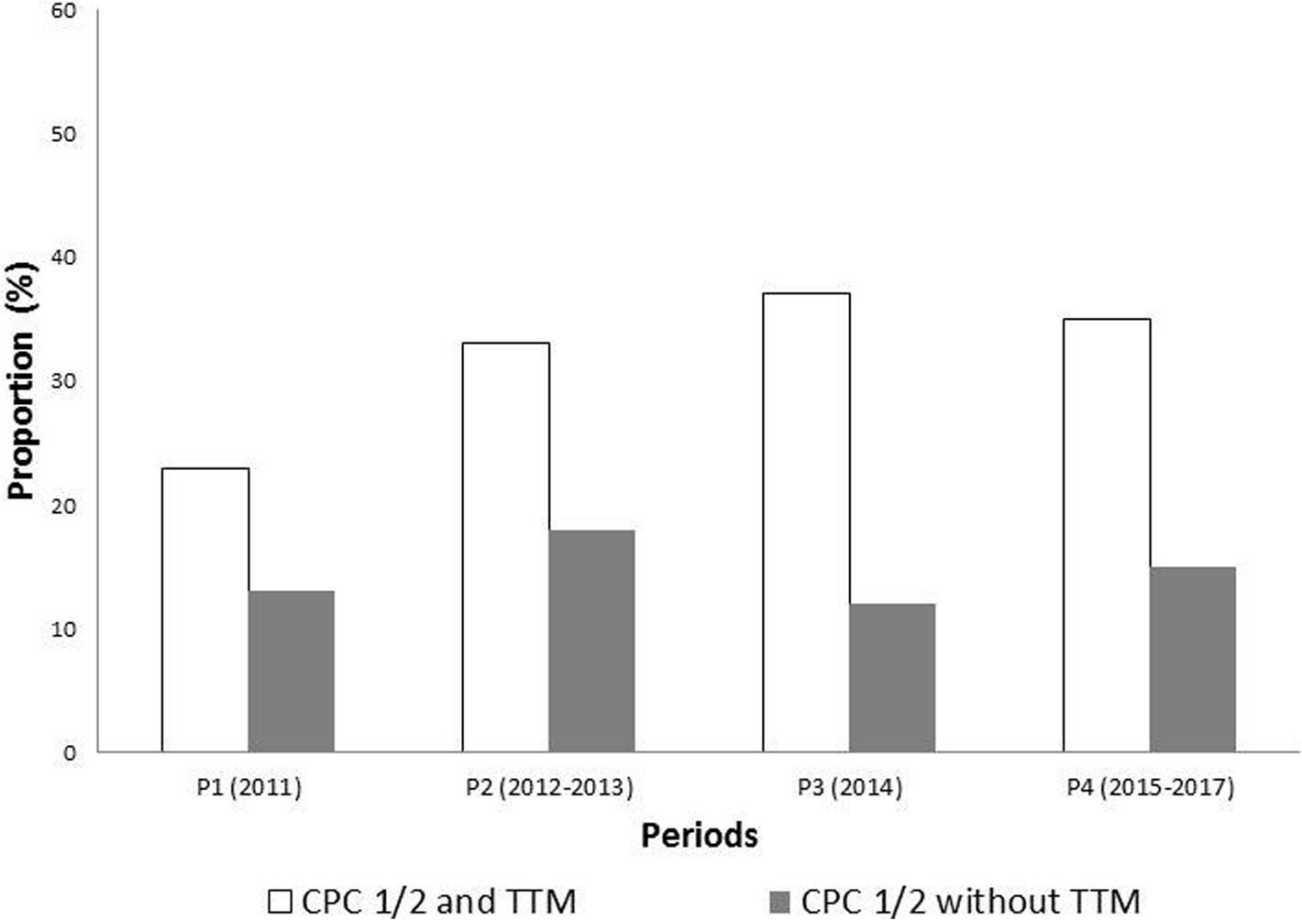
Changes in neurological outcome according to periods of segmented regression analysis

Using segmented regression separating the study period into four separate sub-periods, the association between neurological outcome and periods was significant only for P4 vs. P3 (OR = 0.77 [0.62–0.95]; *P* = 0.015) (see Figs. 2 and 3).

## Discussion

Using the Paris-SDEC registry, which covers a large geographic area in France, we report that the overall proportion of post-cardiac arrest patients who received TTM decreased over years between 2011 and 2017. This decrease was observed in all patients’ subgroups. In parallel, we observed that the activation of early resuscitation improved over time as reflected by an increase in the proportion of patients who received bystander CPR and a decrease in the proportion of patients with a long “no flow.” Despite these improvements in early management, there was no significant change in neurological outcome over the study period.

Recent doubts regarding the efficacy of targeted temperature management (TTM) may have resulted in a loss of interest for this treatment in comatose cardiac arrest (CA) patients. Indeed, following the publication of the TTM trial in 2013, several observational studies reported a change in ICU policies regarding temperature management after cardiac arrest. Up to one third of intensivists changed their temperature target from 33 to 36 °C [6]. Using data from France (where therapeutic hypothermia is commonly used [19]), we report a clear fall in the use of TTM that was not associated with a change in neurological outcome over the same period. In a first approach, these findings do not suggest a detrimental effect of a lesser use of TTM on outcome in this population. However, this could have been concealed by a parallel improvement in the chain of survival that may have masked a negative effect of the decrease in the use of TTM. This interpretation is supported by the protective effect of TTM that was observed in multivariable analysis and also by the segmented regression, which reveals a change in the association between neurological outcome and periods for the most recent years. On the whole, it is not possible to exclude that an improvement in outcome may have been observed if TTM had been more widely used. Of course, the design of the present study does not allow giving any firm conclusion regarding this debate.

The existence of an association between changes in TTM policies and outcome is debated as previous authors reported conflicting results [20, 21]. Our results are in accordance with those from a recent North-American study [8], which explored a quite similar population of post-cardiac arrest patients. On the opposite, our results are discordant with those from Salter et al. [5], who reported an association between the progressive switch from 33 to 36 °C and an increase in both the proportion with fever in ICU and hospital mortality. However, there was no adjustment for major Utstein style confounders in the analysis. In the present study, we observed an increase over the years in bystander CPR and a decrease in no-flow duration, which are both major determinants of outcome [5]. We strongly believe that any further studies aiming to assess the relationship between post-cardiac arrest care and outcome should be adjusted on these major variables.

The loss of confidence in TTM may have been suspected to be deeper in non-shockable patients since the level of evidence regarding its efficacy was lower in this population, as compared with shockable patients [2]. However, a clear insight from the present study is that the lower adherence to TTM after CA was not driven by the cardiac rhythm at EMS presentation, since the decrease was observed in both shockable and non-shockable patients. In several retrospective studies, benefits from TTM at 33 °C were more marked in patients with longer no-flow or low-flow times [22–24], but this result was not replicated in a post hoc analysis of the TTM trial [25]. In this context, it is unfortunate to observe that the fall in TTM use is also observed in shockable patients despite a strong recommendation in recent guidelines [16]. Regarding the subgroup of non-shockable patients, TTM use might increase in the next future due to the results of the HYPERION trial, which showed a significant improvement in neurological outcome at day 90 with TTM at 33° as compared with normothermia [10].

The results of the present study suggest that the TTM trial may have been inaccurately interpreted as a negative study and may have discouraged the use of therapeutic hypothermia in post-cardiac patients. Even if the TTM trial was not designed to assess the efficacy of therapeutic hypothermia, the lack of difference in neurological outcome between patients managed at 33° versus 36° was probably considered as a negative signal regarding the efficacy of cooling. In the meantime, pivotal studies that established the efficacy of TH in post-cardiac arrest patients were challenged in several aspects (low number of patients, highly selected population, temperature strategy in the control group) [26, 27]. Since cooling is a time-consuming treatment, this may explain the disaffection for this treatment. In this way, a particular attention should be paid to the results of the TTM-2 study, which is a large multicentric pragmatic trial comparing moderate hypothermia and normothermia in comatose cardiac patients [28].

Our results should be considered in the context of the study’s limitations. We were unable to comment on potential changes in the quality of therapeutic hypothermia (e.g., time to target temperature and time in target temperature) that may also impact patients’ outcome [29]. The higher proportion of patients with a short no-flow duration is in sharp contrast to prior studies [30], but this proportion is close to what was reported in a non-selected north-American registry [31]. In addition, we decided to study the specific population of patients with a stable ROSC after hospital and ICU admission, a population in which the proportion of shockable patients is known to be high [32]. We were not able to identify the subset of patients who were managed with a strategy of “normothermia or avoiding fever” among those who did not receive TTM. However, each patient’s chart was evaluated by a centralized monitor who checked the medical file for accuracy and competitiveness. Patients in the TTM group were younger, and even after multivariate adjustment, other unmeasured confounders may potentially explain their better outcome. Finally, only short-term outcome was considered, while long-term outcome would have been more adapted (such as 1-month and 3-month outcome as recommended in COSCA guidelines [33]) and we cannot exclude than some non-survivors died of non-neurological cause [34].

## Conclusion

Using a large regional registry of cardiac arrest, we report a progressive decrease in the use of TTM in post-cardiac arrest patients over the recent years. In parallel, we observed that the activation of early resuscitation improved over time as reflected by an increase in the proportion of patients who received bystander-initiated resuscitation and a shorter “no flow.” Despite these improvements in early management, there was no significant change in neurological outcome over the study period. Further research is required to explore the consequence of this decrease in the use of TTM.

## Supplementary information

**Additional file 1.** Patient flowchart.

**Additional file 2.** Proportion of missing values in the study population.

## Data Availability

Data are available on reasonable request to the first and last authors and after approval by all authors of the manuscript.

## Abbreviations

CA: Cardiac arrest
CPR: Cardiopulmonary resuscitation
ROSC: Return of spontaneous circulation
TTM: Targeted temperature management

## References

[1] Kragholm Kristian, Wissenberg Mads, Mortensen Rikke N., Hansen Steen M., Malta Hansen Carolina, Thorsteinsson Kristinn, Rajan Shahzleen, Lippert Freddy, Folke Fredrik, Gislason Gunnar, Køber Lars, Fonager Kirsten, Jensen Svend E., Gerds Thomas A., Torp-Pedersen Christian, Rasmussen Bodil S. (2017). Bystander Efforts and 1-Year Outcomes in Out-of-Hospital Cardiac Arrest. New England Journal of Medicine.

[2] Dumas F, Grimaldi D, Zuber B, Fichet J, Charpentier J, Pene F, Vivien B, Varenne O, Carli P, Jouven X (2011). Is hypothermia after cardiac arrest effective in both shockable and nonshockable patients? Insights from a large registry. Circulation.

[3] Nielsen N, Wetterslev J, Cronberg T, Erlinge D, Gasche Y, Hassager C, Horn J, Hovdenes J, Kjaergaard J, Kuiper M (2013). Targeted temperature management at 33 degrees C versus 36 degrees C after cardiac arrest. N Engl J Med.

[4] Nielsen N, Friberg H, Gluud C, Herlitz J, Wetterslev J (2011). Hypothermia after cardiac arrest should be further evaluated--a systematic review of randomised trials with meta-analysis and trial sequential analysis. Int J Cardiol.

[5] Salter R, Bailey M, Bellomo R, Eastwood G, Goodwin A, Nielsen N, Pilcher D, Nichol A, Saxena M, Shehabi Y (2018). Changes in temperature management of cardiac arrest patients following publication of the target temperature management trial. Crit Care Med.

[6] Deye N, Vincent F, Michel P, Ehrmann S, Silva D, Piagnerelli M, Kimmoun A, Hamzaoui O, Lacherade J-C, Jonghe B (2016). Changes in cardiac arrest patients’ temperature management after the 2013 “TTM” trial: results from an international survey. Ann Intensive Care.

[7] Bray JE, Stub D, Bloom JE, Segan L, Mitra B, Smith K, Finn J, Bernard S (2017). Changing target temperature from 33 degrees C to 36 degrees C in the ICU management of out-of-hospital cardiac arrest: a before and after study. Resuscitation.

[8] Bradley SM, Liu W, McNally B (2018). Temporal trends in the use of therapeutic hypothermia for out-of-hospital cardiac arrest. JAMA Netw Open.

[9] Khera R, Humbert A, Leroux B, Nichol G, Kudenchuk P, Scales D, Baker A, Austin M, Newgard CD, Radecki R (2018). Hospital variation in the utilization and implementation of targeted temperature management in out-of-hospital cardiac arrest. Circulation.

[10] Lascarrou J-B, Merdji H, Le Gouge A, Colin G, Grillet G, Girardie P, Coupez E, Dequin P-F, Cariou A, Boulain T, et al. Targeted temperature management for cardiac arrest with nonshockable rhythm. N Engl J Med. 2019. 10.1056/NEJMoa1906661. 10.1056/NEJMoa1906661 31577396

[11] Bougouin W, Lamhaut L, Marijon E, Jost D, Dumas F, Deye N, Beganton F, Empana JP, Chazelle E, Cariou A (2014). Characteristics and prognosis of sudden cardiac death in Greater Paris: population-based approach from the Paris Sudden Death Expertise Center (Paris-SDEC). Intensive Care Med.

[12] Maupain C, Bougouin W, Lamhaut L, Deye N, Diehl JL, Geri G, Perier MC, Beganton F, Marijon E, Jouven X (2016). The CAHP (Cardiac Arrest Hospital Prognosis) score: a tool for risk stratification after out-of-hospital cardiac arrest. Eur Heart J.

[13] Bougouin W, Dumas F, Karam N, Maupain C, Marijon E, Lamhaut L, Jost D, Geri G, Beganton F, Varenne O (2018). Should we perform an immediate coronary angiogram in all patients after cardiac arrest?: insights from a large French registry. JACC Cardiovasc Interv.

[14] Perkins GD, Jacobs IG, Nadkarni VM, Berg RA, Bhanji F, Biarent D, Bossaert LL, Brett SJ, Chamberlain D, de Caen AR, et al. Cardiac arrest and cardiopulmonary resuscitation outcome reports: update of the Utstein Resuscitation Registry Templates for Out-of-Hospital Cardiac Arrest: a statement for healthcare professionals from a task force of the International Liaison Committee on Resuscitation (American Heart Association, European Resuscitation Council, Australian and New Zealand Council on Resuscitation, Heart and Stroke Foundation of Canada, InterAmerican Heart Foundation, Resuscitation Council of Southern Africa, Resuscitation Council of Asia); and the American Heart Association Emergency Cardiovascular Care Committee and the Council on Cardiopulmonary, Critical Care, Perioperative and Resuscitation. Circulation. 2015;132(13):1286–1300. 10.1161/CIR.0000000000000144 25391522

[15] Jennett B, Bond M (1975). Assessment of outcome after severe brain damage: a practical scale. Lancet.

[16] Nolan JP, Soar J, Cariou A, Cronberg T, Moulaert VR, Deakin CD, Bottiger BW, Friberg H, Sunde K, Sandroni C (2015). European Resuscitation Council and European Society of Intensive Care Medicine Guidelines for Post-resuscitation Care 2015: Section 5 of the European Resuscitation Council Guidelines for Resuscitation 2015. Resuscitation.

[17] White IR, Royston P, Wood AM (2011). Multiple imputation using chained equations: issues and guidance for practice. Stat Med.

[18] Wagner AK, Soumerai SB, Zhang F, Ross-Degnan D (2002). Segmented regression analysis of interrupted time series studies in medication use research. J Clin Pharm Ther.

[19] Orban JC, Cattet F, Lefrant JY, Leone M, Jaber S, Constantin JM, Allaouchiche B, Ichai C (2012). The practice of therapeutic hypothermia after cardiac arrest in France: a national survey. PLoS One.

[20] Casamento A, Minson A, Radford S, Martensson J, Ridgeon E, Young P, Bellomo R (2016). A comparison of therapeutic hypothermia and strict therapeutic normothermia after cardiac arrest. Resuscitation.

[21] Arvidsson L., Lindgren S., Martinell L., Lundin S., Rylander C. (2017). Target temperature 34 vs. 36°C after out-of-hospital cardiac arrest - a retrospective observational study. Acta Anaesthesiologica Scandinavica.

[22] Testori C, Sterz F, Holzer M, Losert H, Arrich J, Herkner H, Krizanac D, Wallmuller C, Stratil P, Schober A (2012). The beneficial effect of mild therapeutic hypothermia depends on the time of complete circulatory standstill in patients with cardiac arrest. Resuscitation.

[23] Kagawa E, Inoue I, Kawagoe T, Ishihara M, Shimatani Y, Kurisu S, Nakama Y, Dai K, Otani T, Ikenaga H (2010). Who benefits most from mild therapeutic hypothermia in coronary intervention era? A retrospective and propensity-matched study. Critical Care (London, England).

[24] Drennan IR, Lin S, Thorpe KE, Morrison LJ (2014). The effect of time to defibrillation and targeted temperature management on functional survival after out-of-hospital cardiac arrest. Resuscitation.

[25] Kjaergaard J, Nielsen N, Winther-Jensen M, Wanscher M, Pellis T, Kuiper M, Hartvig Thomsen J, Wetterslev J, Cronberg T, Bro-Jeppesen J (2015). Impact of time to return of spontaneous circulation on neuroprotective effect of targeted temperature management at 33 or 36 degrees in comatose survivors of out-of hospital cardiac arrest. Resuscitation.

[26] Group HaCAS (2002). Mild therapeutic hypothermia to improve the neurologic outcome after cardiac arrest. N Engl J Med.

[27] Bernard SA, Gray TW, Buist MD, Jones BM, Silvester W, Gutteridge G, Smith K (2002). Treatment of comatose survivors of out-of-hospital cardiac arrest with induced hypothermia. N Engl J Med.

[28] Dankiewicz J, Cronberg T, Lilja G, Jakobsen JC, Bělohlávek J, Callaway C, Cariou A, Eastwood G, Erlinge D, Hovdenes J *et al*: Targeted hypothermia versus targeted normothermia after out-of-hospital cardiac arrest (TTM2). A randomized clinical trial – rationale and design. Am Heart J 2019. 10.1016/j.ahj.2019.06.012 31473324

[29] Deye N, Cariou A, Girardie P, Pichon N, Megarbane B, Midez P, Tonnelier JM, Boulain T, Outin H, Delahaye A, et al. Endovascular versus external targeted temperature management for out-of-hospital cardiac arrest patients: a randomized controlled study. Circulation. 2015. 10.1161/CIRCULATIONAHA.114.012805 26092673

[30] Wissenberg M, Lippert FK, Folke F, Weeke P, Hansen CM, Christensen EF, Jans H, Hansen PA, Lang-Jensen T, Olesen JB (2013). Association of national initiatives to improve cardiac arrest management with rates of bystander intervention and patient survival after out-of-hospital cardiac arrest. Jama.

[31] van Diepen S, Girotra S, Abella BS, Becker LB, Bobrow BJ, Chan PS, Fahrenbruch C, Granger CB, Jollis JG, McNally B *et al*: Multistate 5-year initiative to improve care for out-of-hospital cardiac arrest: primary results from the HeartRescue Project. J Am Heart Assoc. 2017;6(9). 10.1161/JAHA.117.005716 PMC5634254 28939711

[32] Chocron R, Bougouin W, Beganton F, Juvin P, Loeb T, Adnet F, Lecarpentier E, Lamhaut L, Jost D, Marijon E (2017). Are characteristics of hospitals associated with outcome after cardiac arrest? Insights from the Great Paris registry. Resuscitation.

[33] Haywood K, Whitehead L, Nadkarni VM, Achana F, Beesems S, Böttiger BW, Brooks A, Castrén M, Ong MEH, Hazinski MF (2018). COSCA (core outcome set for cardiac arrest) in adults: an advisory statement from the international liaison committee on resuscitation. Resuscitation.

[34] Taccone FS, Horn J, Storm C, Cariou A, Sandroni C, Friberg H, Hoedemaekers CA, Oddo M (2019). Death after awakening from post-anoxic coma: the “Best CPC” project. Crit Care.

