# Supplementary figures and images for: Temporal trends in the use of targeted temperature management after cardiac arrest and association with outcome: insights from the Paris Sudden Death Expertise Centre
Source: Crit Care. 2019 Dec 3;23:391. doi: 10.1186/s13054-019-2677-1 (PMC6892202; doi:10.1186/s13054-019-2677-1)

Figure S1: Patient flowchart
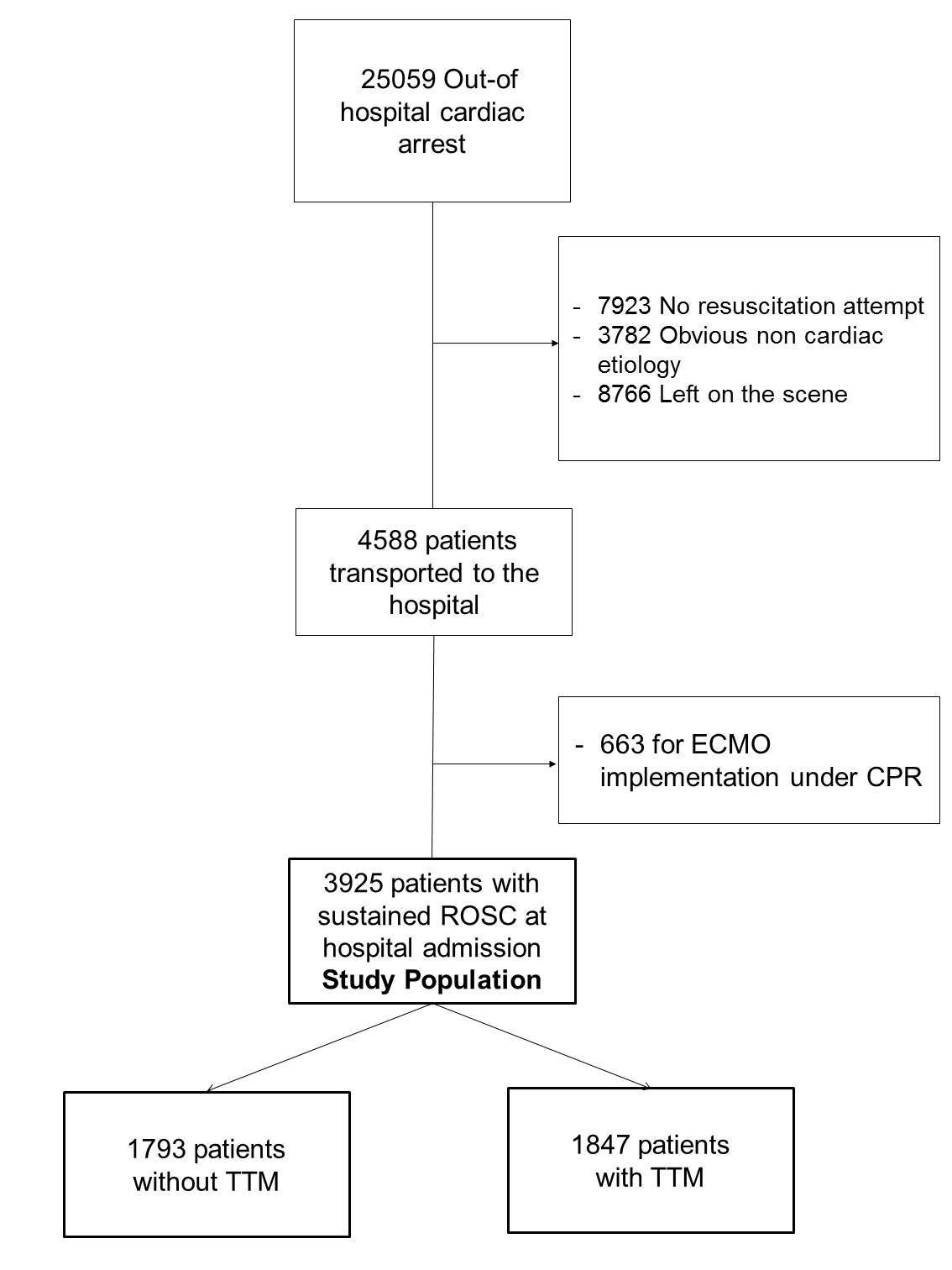

Supplement: Supplementary file 1 — Additional file 1. Patient flowchart. [file 13054_2019_2677_MOESM1_ESM.docx]
