# Supplementary material for: Temporal trends in the use of targeted temperature management after cardiac arrest and association with outcome: insights from the Paris Sudden Death Expertise Centre
Source: Crit Care. 2019 Dec 3;23:391. doi: 10.1186/s13054-019-2677-1 (PMC6892202; doi:10.1186/s13054-019-2677-1)
Supplement: Supplementary file 2 — Additional file 2. Proportion of missing values in the study population. [file 13054_2019_2677_MOESM2_ESM.docx]

Table S1 : Proportion of missing values in the study population (N=3925)

|  | Missing |
| --- | --- |
| Male, n (%) | 1  (<1%) |
| Age, n (%) | 4  (<1%) |
| Home location, n (%) | 7  (<1%) |
| Witnessed, n (%) | 30  (<1%) |
| Bystander CPR before EMS arrival, n (%) | 417  (10%) |
| Initial shockable rhythm, n (%) | 68  (2%) |
| No flow > 3 min*, n (%) | 595  (15%) |
| Low flow > 20 min*, n (%) | 794  (20%) |
| Use of epinephrine, n (%) | 163  (4%) |
| First arterial pH, n (%) | 1011  (25%) |
| Post-resuscitation shock, n (%) | 566  (14%) |
| Early invasive coronary strategy, n (%) | 193  (5%) |
| Targeted temperature management, n (%) | 285  (7%) |
| Survival at ICU discharge, n (%) | 156  (4%) |
| Good neurological prognosis at ICU discharge, n (%) | 302  (8%) |

* Characteristics were dichotomized according to the median value
